# The Contribution of Hippocampal All-Trans Retinoic Acid (ATRA) Deficiency to Alzheimer’s Disease: A Narrative Overview of ATRA-Dependent Gene Expression in Post-Mortem Hippocampal Tissue

**DOI:** 10.3390/antiox12111921

**Published:** 2023-10-27

**Authors:** Joey Almaguer, Ashly Hindle, J. Josh Lawrence

**Affiliations:** 1 Department of Pharmacology and Neuroscience, Texas Tech University Health Sciences Center, Lubbock, TX 79430, USA; joey.almaguer@ttuhsc.edu; 2 Department of Pharmacology and Neuroscience and Garrison Institute on Aging, Texas Tech University Health Sciences Center, Lubbock, TX 79430, USA; ashly.hindle@ttuhsc.edu; 3 Department of Pharmacology and Neuroscience, Garrison Institute on Aging, Center of Excellence for Translational Neuroscience and Therapeutics, and Center of Excellence for Integrated Health, Texas Tech University Health Sciences Center, Lubbock, TX 79430, USA

**Keywords:** retinoic acid, vitamin A, Alzheimer’s disease, aging

## Abstract

There is accumulating evidence that vitamin A (VA) deficiency contributes to the pathogenesis and progression of Alzheimer’s disease (AD). All-*trans* retinoic acid (ATRA), a metabolite of VA in the brain, serves distinct roles in the human hippocampus. Agonists of retinoic acid receptors (RAR), including ATRA, promote activation of the non-amyloidogenic pathway by enhancing expression of α-secretases, providing a mechanistic basis for delaying/preventing amyloid beta (Aβ) toxicity. However, whether ATRA is actually deficient in the hippocampi of patients with AD is not clear. Here, using a publicly available human transcriptomic dataset, we evaluated the extent to which ATRA-sensitive genes are dysregulated in hippocampal tissue from post-mortem AD brains, relative to age-matched controls. Consistent with ATRA deficiency, we found significant dysregulation of many ATRA-sensitive genes and significant upregulation of RAR co-repressors, supporting the idea of transcriptional repression of ATRA-mediated signaling. Consistent with oxidative stress and neuroinflammation, Nrf2 and NfkB transcripts were upregulated, respectively. Interestingly, transcriptional targets of Nrf2 were not upregulated, accompanied by upregulation of several histone deacetylases. Overall, our investigation of ATRA-sensitive genes in the human hippocampus bolsters the scientific premise of ATRA depletion in AD and that epigenetic factors should be considered and addressed as part of VA supplementation.

## 1. Introduction

Approximately 6.7 million Americans are currently living with Alzheimer’s disease (AD) [1]. As the aging US population grows, 13.8 million Americans are projected to have AD by 2060 [1]. Currently, two treatments for AD are approved by the Food and Drug Administration, but there are no cures that are capable of reversing or halting the neurodegenerative progression of AD. Genetic predisposition and age are unmodifiable risk factors for AD. However, several environmental and lifestyle risk factors, such as diet, smoking, and physical activity, are modifiable. Poor diet is a leading cause of death in the US and worldwide [2,3], and is associated with a number of comorbidities (e.g., cardiovascular disease, obesity, diabetes) that increase the risk for AD [4,5,6,7,8]. Observational studies and clinical trials have demonstrated the protective benefits of a healthy diet in relation to AD pathogenesis [9,10]. Moreover, dietary intake of vegetables is associated with high levels of serum antioxidants (AOs) and reduced Aβ accumulation [11].

It is widely accepted that the toxic accumulation of amyloid beta (Aβ) and neurofibrillary tangles leads to progressive cognitive decline, ultimately culminating in AD-induced death [12]. The Mitochondrial Free Radical Theory of Aging proposes that reactive oxygen species (ROS), produced as a product of normal mitochondrial metabolism, cause oxidative damage to proteins, lipids, and nucleic acids which accumulates across the lifespan [13]. Antioxidants (AOs), both diet-derived, and Nrf2-mediated endogenous AOs, help prevent ROS-induced damage. The condition in which cellular AO capacity is insufficient to neutralize ROS is termed oxidative stress (OS). Therefore, the preconditions necessary for ROS-induced oxidative damage require not only ROS generation, but also the depletion of dietary and/or endogenous AO defenses. AO replenishment is an intuitive therapeutic strategy to attenuate, delay, or altogether prevent age-related cognitive decline (ARCD) and AD. Paradoxically, the use of generic ROS scavengers, such as vitamin E, as AO monotherapies have failed in clinical trials [14,15,16]. These failures have revealed that faulty assumptions and major knowledge gaps exist in our models of the biological etiology and mechanisms of AD.

We hypothesize that the progression of AD may involve deficiencies in specific AOs that have roles beyond that of generic ROS scavengers. In this study, we highlight the multifaceted roles of all-trans retinoic acid (ATRA) with particular emphasis on the growing evidence of ATRA deficiency in AD. Moreover, we present transcriptomic evidence of ATRA deficiency in human AD reflecting its in vivo roles as both a transcription factor and an AO.

## 2. Results and Discussion

### 2.1. ATRA Transport and Metabolism

**Figure 1:**
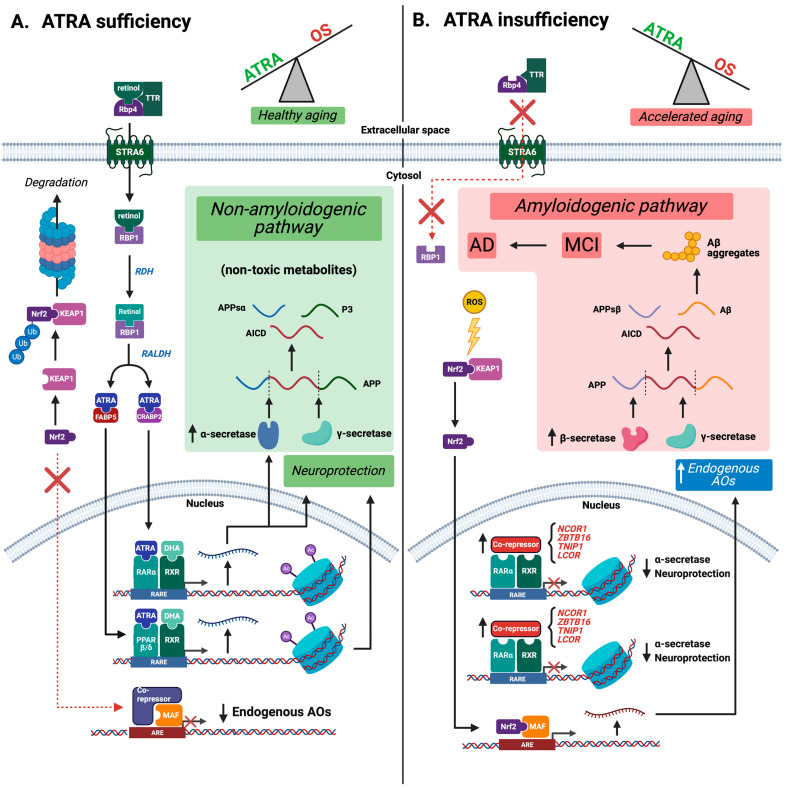
Distinct roles of ATRA and Nrf2-mediated AO defenses in the hippocampus proposed during normal aging and AD. (**A**) The non-amyloidogenic pathway and the transcription of neuroprotective proteins is promoted by ATRA suffiency, in coordination with the RXR agonist DHA. (**B**) The amyloidogenic pathway is promoted by ATRA deficiency. The subsequent rise in ROS causes oxidation of the Nrf2-KEAP1 complex, resulting in a compensational increase in endogenous AO defenses.

Under healthy physiological conditions, ATRA sufficiency promotes health through the scavenging of excess ROS and by inducing the expression of genes critical for synaptic function and neuroprotection (Figure 1A). Retinol (a form of VA) is first transported from liver stores to the hippocampus through the bloodstream, bound to a carrier complex that consists of retinol binding protein 4 (RBP4) [17] and transthyretin (TTR) [18]. Retinol crosses the blood brain barrier, facilitated via RBP4 binding to its receptor, signaling receptor and transporter of retinol STRA6 (STRA6) [19]. Retinol also enters neurons via STRA6, which catalytically dissociates retinol from RBP4 and transports it across the plasma membrane [19]. Once within the cytoplasm, retinol binds to cellular retinol binding protein 1 (RBP1) and is converted into retinal by one of several retinol dehydrogenase enzymes (RDH). RBP1-bound retinal is then converted into ATRA by a retinaldehyde dehydrogenase enzyme (ALDH1a1/2/3) and is transferred to several binding proteins: cellular retinoic acid binding protein 1/2 (CRABP1/2) or fatty acid binding protein 5 (FABP5). As shown in cell lines, the binding of ATRA to either CRABP1/2 or FABP5 is a defining step because it determines nuclear receptor localization and, subsequently, which genes are transcriptionally activated [20,21,22].

### 2.2. Cellular Binding Proteins Traffic ATRA to Distinct Nuclear Receptors, Collectively Promoting Activation of the Nonamyloidogenic Pathway

When bound to CRABP2, ATRA is transported into the nucleus and transferred to retinoic acid receptor (RARs), as has been shown in astrocytes [23,24]. ATRA-RARα becomes heterodimerized with the retinoid X receptor (RXR). The ATRA-RARα/RXR complex enables activation of transcription, as RXR is a non-permissive partner [25]. However, RXR can also bind many co-agonists, including docosahexaenoic acid (DHA), 9-cis-RA, and others [26,27,28]. ATRA binding to RARα/RXR results in the dissociation of bound co-repressors and recruits transcriptional activators and histone acetyltransferases (HATs). This heterodimer binds to promoter retinoic acid response elements (RAREs) to increase the transcription of ATRA-responsive genes, including the α-secretase ADAM10 [29] which mediates processing of amyloid precursor protein (APP) into non-amyloidogenic fragments, as well as additional genes involved in synaptic plasticity that are neuroprotective against AD pathogenesis. Unlike the toxic amyloid-β peptides that result from β-secretase (BACE1) cleavage, these cleavage products do not aggregate to form plaques or catalyze the aberrant formation of ROS (Figure 1A).

When bound to FABP5, ATRA is transported into the nucleus and transferred to peroxisome proliferator-activated receptor β/δ (PPARβ/δ), which also dimerizes with RXRs [30]. The PPARβ/δ-RXR heterodimer then binds to peroxisome proliferator response elements (PPREs) to express genes important for neuroprotection [31,32,33,34] (Figure 1A). ATRA binding to PPARβ/δ-RXR induces transcription of genes that function to minimize OS, neuroinflammation, and neurotoxicity, and to decrease Aβ and hyperphosphorylated tau (p-tau) levels [30,35,36,37,38]. PPARβ/δ knockout mice exhibit increased expression of β-secretase (BACE1), receptor for advanced glycation endproducts (RAGE), proinflammatory and proapoptotic mediators, and tau hyperphosphorylation [30,35], implicating PPARβ/δ receptors in AD pathology. Mice treated with GW0742, a selective PPARβ/δ agonist, showed a reduction in parenchymal Aβ plaque deposition, diminished neuroinflammatory and apoptotic states, and decreased activation of microglia [39,40,41]. Similarly, T3D-959, a synthetic agonist of both PPARβ/δ and PPARγ, with 15-fold higher selectivity for PPARβ/δ, has shown promise in clinical trials [42,43,44]. Other PPARs outside of PPARβ/δ, such as PPARα and PPARγ, have also been linked to neuroprotection against AD [45,46].

Similarly to the ATRA-mediated activation of RARα, the activation of PPARα promotes the non-amyloidogenic processing of APP by inducing the transcription of the α-secretase ADAM10 [47,48,49]. Activation of PPARγ results in decreased levels of neuroinflammation, OS, and Aβ; however thiazolidinedione drugs which are agonists for PPARγ have not shown promise in clinical trials due to poor BBB penetration [50]. Interestingly, both PPARα and PPARβ/δ are downregulated in AD brains, whereas PPARγ is upregulated [51]. Although PPARα and PPARγ are not directly activated by ATRA, their dimer partner RXR is activated by 9-cis-retinoic acid [52,53], an isomerization product of ATRA, suggesting a mechanism by which ATRA and/or retinol levels may affect these pathways.

Transcription of RARα- and PPARβ/δ-sensitive genes is dependent on the levels of CRABP2 and FABP5, respectively [21,22]. Increased expression of CRABP2 yields higher transcription of RARα-regulated genes, whereas increased expression of FABP5 yields increased expression of PPARβ/δ-regulated genes [21,54]. ATRA has a similar binding affinity for CRABP2 and FABP5, though the agonism depends on competing fatty acids [20]. Therefore, many factors depend on how the two pathways are tuned to be differentially responsive to different ATRA concentrations, suggesting that the key regulatory activities of each pathway are relevant under distinct physiological states [31]. Additionally, PPARβ/δ polymorphisms in patients do not seem to be significant risk factors for AD, arguing against a central role of ATRA-PPARβ/δ in brain neuroprotection [55,56]. In summary, ATRA-responsive RARα and PPARβ/δ pathways appear to converge on mechanisms which reduce OS and Aβ accumulation, which should, in principle, delay AD onset and progression. If ATRA concentrations remain too low for significant binding interactions to occur, then PPARβ/δ polymorphisms would not be expected to influence AD progression. However, PPARβ/δ agonists may still be able to provide neuroprotective benefits in patients treated with PPARβ/δ agonists or retinol supplementation.

### 2.3. The Role of ATRA in AD Animal Models

Our current understanding of the role ATRA in the hippocampus of rodent AD models come from six lines of converging evidence. First, VA plays an important role in hippocampal-dependent learning. VA deficiency in the brain is associated with impairment in hippocampal-dependent learning [57,58,59,60]. In contrast, ATRA sufficiency stimulates neurogenesis, hippocampal dendritic growth, and hippocampal learning and memory operations [57,61,62]. Second, ATRA deficiency increases Aβ levels in the normal rat brain by decreasing expression of the α-secretase enzyme ADAM metallopeptidase domain 10 (ADAM10), disrupting the balance between non-amyloidogenic and amyloidogenic APP processing pathways [63,64]. Moreover, direct activation of RARs upregulates ADAM10 and promotes activation of the non-amyloidogenic pathway, reducing Aβ generation and deposition [65,66,67]. Third, ATRA or retinol supplementation can restore hippocampal ATRA levels, preserve learning capability, rescue hippocampal-dependent learning impairment, and reduce amyloid load in AD mouse models [57,68,69]. Fourth, even when liver stores of VA are sufficient, hippocampal ATRA levels are dramatically reduced with hepatic inflammation and/or age, suggesting liver-brain dysregulation in the hepatic release, vascular transport, or hippocampal processing of VA [57,59,70]. Fifth, chronic neuroinflammation in microglia upregulates the expression of CYP26, an enzyme involved in ATRA degradation, implying that chronic neuroinflammation, as observed in AD, is associated with reduced hippocampal ATRA levels [71]. Treatment with ATRA or a RARα agonist exerts potent anti-inflammatory effects [68,71,72,73]. Finally, ATRA plays a non-genomic role in homeostatic plasticity by balancing excitatory and inhibitory synaptic strength, mediated by postsynaptic calcium-dependent regulation of ATRA synthesis [74,75,76,77]. In contrast, RARα conditional knockout mice, which are deficient in ATRA signaling in the hippocampal CA1 region, display runaway Hebbian synaptic plasticity [78].

### 2.4. The Role of ATRA in Human AD

There is growing evidence for a role of ATRA in the human hippocampus with regards to cognitive function [79]. First, there is a negative correlation between retinol/carotenoid intake or serum levels and AD-related cognitive decline [11,80,81,82,83,84,85,86,87,88,89,90,91,92,93,94]. Second, there is a negative correlation between serum retinol/carotenoid levels and biomarkers of OS [95,96]. On the other hand, a positive correlation has been shown between serum carotenoids levels and telomere length in leukocytes, which is considered a biomarker of heathy aging [84,97]. Third, genes involved in retinol metabolism and function have been linked to late-onset AD [98,99,100]. Moreover, loss of function mutations in the α-secretase ADAM10 result in seizures, further strengthening the association between AD and epilepsy [101,102]. Consistent with this association, ADAM10 overexpression suppresses seizures and inflammation in epilepsy animal models [103]. Fourth, VA/ATRA dysregulation is associated with environmentally induced comorbidities that are now recognized as leading risk factors for AD, including cardiovascular disease, obesity, hepatosteatosis, and diabetes [104,105]. Lastly, a randomized control trial demonstrated that treatment with the RAR agonist isotretinoin increased activation of the nonamyloidogenic pathway, as indicated by increased cerebrospinal fluid levels of APPsα, a neuroprotective cleavage product of APP [106].

### 2.5. A Critical Knowledge Gap: ATRA-Sensitive Gene Expression in Postmortem Human AD Hippocampus

**Figure 2:**
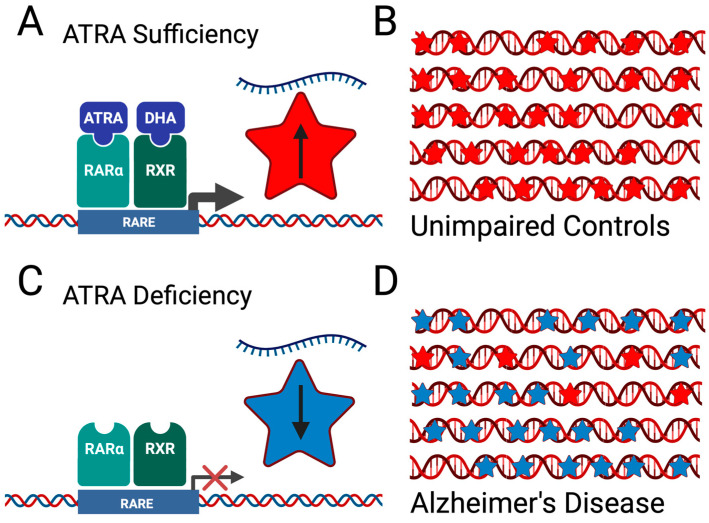
Theoretical detection of ATRA deficiency through gene expression analysis of human brain transcriptomics data. (**A**) Activation of RAR-RXR heterodimers induces increased transcription of genes that possess retinoic acid response elements (RAREs; red star). (**B**) In the case of ATRA sufficiency in unimpaired control brains, transcription is maintained across ATRA-sensitive genes (red stars). (**C**) Deficiency in ATRA leads to transcriptional repression of ATRA-sensitive genes (blue star). (**D**) In the case of ATRA deficiency in Alzheimer’s disease brains, transcription is decreased across ATRA-sensitive genes (blue stars).

Despite the considerable amount of correlative evidence for ATRA deficiency in AD patients and accompanying hippocampal-related learning deficits, there is currently no direct evidence that ATRA is deficient in the hippocampus of AD patients. We surmised that hippocampal ATRA deficiency could be inferred through the dysregulation of ATRA-sensitive genes, essentially making use of ATRA-sensitive genes as endogenous ATRA sensors (Figure 2). Therefore, using a previously published RNAseq dataset from the hippocampi of postmortem AD and control brains [107], we performed a secondary analysis of publicly available hippocampal transcriptomic data that was generated from 20 AD cases and 10 controls, originally acquired from the Netherlands Brain Bank by van Rooij and colleagues [107]. This data set contains a heterogeneous population of AD hippocampal tissue samples that included both sexes and multiple APOE genotypes [107]. We examined genes known to possess RAREs and/or known to be transcriptionally controlled by ATRA (the VA transcriptome) [108,109,110,111], which included 297 genes that were shown to possess RARβ binding sites [112]. In addition, we also consulted the web-accessible transcription factor databases TF2DNA [113] and Enrichr [114,115,116] for ATRA-sensitive genes and ATRA-related transcription factors.

Collectively, this study provides new evidence that numerous ATRA-sensitive genes are differentially expressed (DE) in AD compared to control hippocampus. We discovered that RAR co-repressors were upregulated, suggesting that, in addition to ATRA deficiency, downstream ATRA-responsive transcription is actively blocked. We also found indirect evidence for ATRA depletion via Nrf1 and Nrf2, master OS-responsive transcription factors. Nrf1 and Nrf2 were upregulated in the hippocampus of human AD brains, consistent with ATRA depletion and its activity as an antioxidant. Interestingly, we discovered that transcriptional targets of Nrf2 were downregulated, providing evidence that even though Nrf2 was upregulated in AD hippocampus, this transcription factor was unable to successfully induce the expression of AO defenses. Finally, we found that several histone deacetylases (HDACs) were upregulated. This finding, along with the finding that Nrf2-responsive genes were downregulated despite Nrf2 upregulation, suggests that epigenetic silencing may interfere with the capability of transcription factors such as RAR and Nrf2 to transactivate their target genes, resulting in the depletion of both exogenous and endogenous antioxidants and collectively contributing to oxidative stress and neurodegeneration. In this review, we discuss some of the transcriptomic findings supporting the dysregulation of retinoid signaling in the hippocampus of AD patients.

### 2.6. Genes Mediating Retinol Transport, ATRA Synthesis, and ATRA Metabolism Are Dysregulated in Hippocampal Tissue from Post-Mortem AD Brains

We first examined the expression of genes involved in retinoid transport and metabolism, as well as retinoid responsive transcription factors (Table 1). Retinol binding protein 4 (RBP4) and transthyretin (TTR) are retinol carriers produced by the liver (Figure 1). Interestingly, RBP4 and TTR transcripts were detected in postmortem hippocampal tissue. Moreover, RBP4 was significantly downregulated in human AD. If hippocampal RBP4 transcript is reflective of the level of RBP4 protein in circulation, the dysregulation of hippocampal RBP4 transcript may signify changes in liver RBP4 expression, suggesting impaired retinol transport along the liver-brain axis. Alternatively, brain and liver RBP4 transcript expression may be completely independent of each other; future research is needed to determine relationships in RBP4 expression between liver and brain. RBP1 (frequently designated CRBP1), the intracellular retinol carrier that accepts retinol from the STRA6 transporter [20], was also found to be transcriptionally downregulated in the hippocampus of AD brains (Table 1). Both RBP1 and RBP4 are known to be transcriptionally regulated by ATRA [108,110,111,117,118]. Retinol dehydrogenase 12 (RDH12) is one of the major cytoplasmic enzymes used in the conversion of retinol to retinal and was significantly downregulated. ADLH1A1, ADLH1A2, and ADLH1A3 are involved in the cytoplasmic conversion of retinal to ATRA. ALDH1A3 was significantly downregulated, suggesting reduced production of ATRA. CRABP2 transfers ATRA to RARα, whereas FABP5 transfers ATRA to PPARβ/δ [31]. CRABP2 and FABP5 transcripts were both detected in the hippocampus but not significantly dysregulated. 

CYP26A1 and CYP26B1 transcripts encode ATRA-metabolizing enzymes. CYP26A1 is known to contain canonical DR5 RARE motifs within its promoter region [111,119], enabling feedback control of ATRA levels. CYP26A1 and CYP26B1 cause hydroxylation of ATRA, which promotes the clearance of ATRA used for intracellular and paracrine homeostatic balancing, as well as the formation of other bioactive retinoids that bind to RAR isoforms with lower but variable affinities: 4-OH-RA, 18-OH-RA, 16-OH-RA, and 4-oxo-RA [120,121]. CYP26A1 and CYP26B1 transcripts were significantly downregulated in AD hippocampus. One simple interpretation of the downregulation of these CYP26 enzymes is that it is likely reflective of ATRA deficiency, as both are induced by ATRA through feedback regulation to protect neurons against excessive accumulation of ATRA [120,122].

Expression levels of several RARs (RARA, RARB) were not significantly changed in the AD condition. However, the interpretation is complicated by the fact that both RARA and RARB have distinct isoforms with different transcription start sites, some of which are directly regulated by RAREs, while some are regulated through indirect means. For example, the promoter sequence regulating the transcripts encoding the RARα1 isoform lacks a RARE, while the promoter regulating expression of the RARα2 isoform does contain a RARE [123,124]. Unfortunately, RNA-sequencing results do not discriminate between these two transcripts. RARG was upregulated. However, RARG is unconventional in that the RARE in the promoter is flanked by SP1 transcription factor binding sites [125]. SP1 has been shown to be upregulated in AD [126], which is also the case here (Table 1). Overall, despite these caveats, we interpret these observations to be generally indicative of ATRA deficiency.

### 2.7. Transcriptional Upregulation of RAR-Related Co-repressor Genes in the Post-mortem Hippocampus in AD

In the absence of ATRA, RAREs are normally repressed by a number of RAR co-repressors, thereby interfering with the transcription of ATRA-sensitive genes [127]. We detected the RAR-related co-repressors NCOR1, NCOR2, ZBTB16, TNIP1, RIF1, and LCOR in postmortem AD hippocampal tissue. Importantly, most of these co-repressors [127,128] were significantly and uniformly upregulated (Table 2), consistent with impaired transcription of RAR-dependent downstream signaling. Nuclear Receptor Corepressor 1 (NCOR1) is involved in ATRA-independent transcriptional repression [129], which complexes with histone deacetylases (HDACs) to promote condensation of chromatin and epigenetic silencing. Similarly, promyelocytic leukemia zinc finger protein (PLZF, currently known as ZBTB16) complexes with RARα, resulting in transcriptional repression likely by preventing RXR-RARα heterodimerization [130,131]. However, ZBTB16 also represses the transcriptional activity of other Class II (RXR heterodimer) nuclear receptors, so it is not restricted to RXR-RARα [131]. Interestingly, TNIP1 is also a co-repressor of RARα and PPARs. However, it atypically represses these nuclear receptors when they are bound to ATRA [128,132]. This observation suggests that TNIP1 upregulation may interfere with ATRA-sensitive transcription of RAR genes [128,132]_._ RIF1 has also been shown to have RAR co-repressor activity, but it is not clear to what extent it has co-repressor activity at other nuclear receptors [133]. We detected a modest upregulation of RIF1, though the significance did not meet the false positive rate threshold. Finally, LCOR is a co-repressor of RARs and other types of nuclear receptors [134], which was significantly upregulated. Generally, the upregulation of RAR-related co-repressors is consistent with transcriptional block of RAR signaling in human AD, corroborating the transcriptional downregulation of ATRA-sensitive genes, collectively converging on a strong scientific premise for ATRA deficiency.

### 2.8. Increased Expression of OS and Neuroinflammatory Genes Is Consistent with ATRA Depletion

Depletion of antioxidants in AD are likely to shift redox balance toward elevated OS [135,136,137]. In addition to being a hormone-like nuclear receptor [138,139], ATRA, as well as Pro-VA carotenoids and retinol, is thought to possess intrinsic antioxidant/ROS scavenging properties [140,141,142]. Therefore, we hypothesize that enhancement of OS biomarkers, as well as compensatory increases in endogenous AO defenses, should accompany the downregulation of ATRA-sensitive genes. We found significantly upregulated transcription of the major OS-responsive transcription factors Nrf1 (NFE2L1) and Nrf2 (NFE2L2). Nrf1/2 bind to antioxidant response elements (AREs) to activate enzymes and molecules involved in endogenous AO defenses [141]. To be consistent with Nrf2-mediated gene expression, these findings should be accompanied by downstream increases in the expression of Nrf1/2 target genes. Interestingly, we found a significant downregulation in transcripts of Nrf2-regulated endogenous AO defense genes (Table 3), including NADPH Quinone oxidoreductase enzyme 1 and 2 (NQO1 and NQO2), glutathione s-transferase alpha 4 (GSTA4), glutathione s-transferase mu 4 (GSTM4), prostaglandin reductase 1 (PTGR1), heme oxygenase 2 (HMOX2), superoxide dismutase type 1 (SOD1), and glutaredoxin (GLRX). These results are consistent with the hypothesis that aberrant AD-related epigenetic silencing may limit the ability of transcription factors such as Nrf2 or RAR/RXR to initiate and/or maintain transcription. This downregulation of Nrf2-mediated endogenous AO defenses was not generally uniform, with catalase (CAT) transcription being modestly upregulated. This observation may indicate that epigenetic silencing in AD is not a uniform process, or that CAT upregulation could have been driven by other transcription factors which act at the promoter of the CAT gene [143]. This latter scenario seems unlikely—it is difficult to envision how silenced promoters would maintain selective responsiveness to only certain transcription factors. 

Together with Nrf1 and Nrf2, the family of homodimeric and heterodimeric transcription factors known as Nuclear Factor Kappa B (NF-κB) regulates and coordinates cellular responses to OS and neuroinflammation. We found that the NF-κB family genes NFKB1 (also called p105) and NFKB2 (also called p52) were upregulated in the hippocampus of AD patients (Table 3), indicative of neuroinflammation and OS seen in the AD condition [144,145]. Proinflammatory cytokines such as Tumor Necrosis Factor-Alpha (TNF, commonly called TNF-α) and Interleukin 1 Beta, (IL1B, commonly called IL-1β), present at elevated levels in the blood of the AD patients, are activators of NF-κB [146]. RELA (also known as p65, also a transcription factor of the NF-κB family), transcriptionally represses ARE-sensitive gene expression by competition with Nrf2 for binding of the CBP/p300 co-activator and by recruiting HDAC3 to AREs [147]. This prevents the production of endogenous antioxidants, further activating NF-κB in a potentially pathological positive-feedback cycle [147]. Furthermore, NF-κB can translocate Keap1 into the nucleus to facilitate Nrf2 degradation and can even regulate Nrf2 expression by binding to its promoter region [148]. On the other hand, Nrf2 regulates NF-κB activity by reducing levels of ROS and inhibiting RAC1-mediated activation of NF-κB [148]. Moreover, studies have shown that ATRA counteracts neuroinflammation by reducing pro-inflammatory cytokines and modulating NF-κB activation, suggesting that upregulation of NF-κB transcription factors, and possibly neuroinflammation in general, are promoted by ATRA deficiency [149,150,151,152,153,154,155,156,157].

### 2.9. Crosstalk between RAR and Nrf2 Signaling

**Figure 3:**
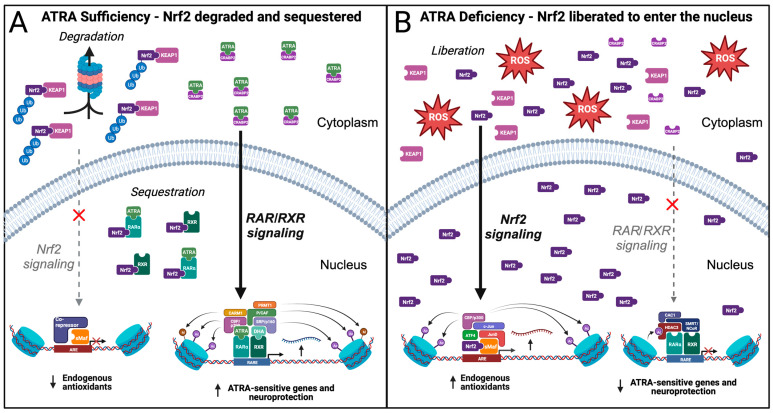
Crosstalk between RAR/RXR and Nrf2 signaling. (**A**) In ATRA sufficiency, a reduced intracellular environment promotes binding of Nrf2 to KEAP1, facilitating degradation of Nrf2. In addition, Nrf2 is sequestered by RARα and RXR receptors. The availability of ATRA leads to sustained transcription of ATRA-sensitive genes, promoting neuroprotection. (**B**) In ATRA deficiency, a relative absence of antioxidants causes the generation of reactive oxygen species (ROS). Oxidative stress causes the liberation of Nrf2 from KEAP1, allowing Nrf2 to enter the nucleus. Within the nucleus, Nrf2 dimerizes with sMaf at antioxidant response elements (AREs), leading to increased transcription of endogenous antioxidants that serve to counteract ROS and ROS-induced damage. The relative absence of ATRA diminishes RAR/RXR-mediated signaling, leading to a downregulation of ATRA-sensitive genes that mediate neuroprotection.

As part of homeostatic balance in the healthy brain, redox balance should be achieved by a combination of exogenous and endogenous antioxidants. Therefore, Nrf2- and RAR signaling pathways should have mechanisms for cross-sensing their activation states. Nrf2 signaling and AO defenses are sensitive to oxidative stress, which we argue may be accompanied by depleted ATRA availability. However, sufficient bidirectional control mechanisms should be in place such that sufficient ATRA bioavailability suppresses Nrf2 signaling in the nucleus by trapping Nrf2 in the cytoplasm through Keap1-Nrf2 interactions, facilitating Nrf2 degradation (Figure 3A). Indeed, there is also evidence that Nrf2 can also be directly sequestered by ATRA-liganded RARα by binding to the Neh7 domain on Nrf2, thereby reducing transcription of Nrf2-mediated AO defenses [158,159]. Similar to RARα agonists, RXRα agonists can repress ARE-dependent gene expression [160]. Moreover, the minimization of ROS accumulation, contributed by ATRA sufficiency, facilitates Keap1-Nrf2 association, which inactivates downstream actions of Nrf2 through subsequent ubiquitination and proteasomal degradation [161]. Interestingly, RXRα has also been shown to sequester Nrf2 without needing to be liganded to ATRA or heterodimerized to RARα [162]. Additionally, it has been shown that in acute myeloid leukemia (AML) cells, ATRA suppress Nrf2 translocation into the nucleus by an unspecified mechanism, further repressing Nrf2-mediated effects [163]. Moreover, ATRA may even directly prevent Nrf2 translocation into the nucleus [163,164], which indicates a complex relationship between exogenous AO sources (i.e., ATRA) and endogenous AOs in maintaining redox balance (Figure 3A). However, further study is required to confirm that this response occurs with neuronal Nrf2. Together, these findings suggest that RAR- and Nrf2-mediated signaling are in homeostatic equilibrium, in which redox balance is in part determined by ATRA availability and cellular expression of Nrf2-mediated endogenous AO defense mechanisms. With ATRA sufficiency, multiple mechanisms exclude Nrf2 from entering the nucleus through direct binding of liganded RARα as well as through Nrf2-Keap1 interactions, ultimately resulting in repressed expression of Nrf2-mediated endogenous AOs. This is not to suggest a complete deactivation of Nrf2-induced expression, rather a coordinated attenuation dependent on hippocampal ATRA concentration which modulates cellular antioxidant mechanisms to counteract ROS accumulation.

By contrast, ATRA deficiency or RARα antagonism promotes Nrf2 binding to AREs [159]. Moreover, the resulting increase in OS mediated by ATRA depletion leads to the dissociation of Nrf2 from Keap1 (Figure 3B). This mechanism occurs via oxidation of the thiol groups (-SH) of cysteine residues on Keap1 by ROS, facilitating the release and translocation of the transcription factor Nrf2 from the cytosol to the nucleus [165]. Nrf2 subsequently dimerizes with small Maf (sMaf) and binds to AREs, inducing the upregulation of endogenous AO pathways that counteract ROS accumulation and limit further oxidative damage to cellular machinery [166,167,168]. Given this intimate association between RARα and Nrf2 signaling, the dysregulation of ATRA-sensitive genes combined with the engagement of Nrf2-sensitive endogenous AO defenses corroborates the idea of ATRA deficiency. In some circumstances, the mechanism of ATRA-induced downregulation of Nrf2 signaling could exacerbate oxidative damage. In the context of leukemia therapy, dismantling AO defenses through ATRA-induced downregulation of Nrf2 signaling can be exploited to help kill cancer cells [163].

**Figure 4:**
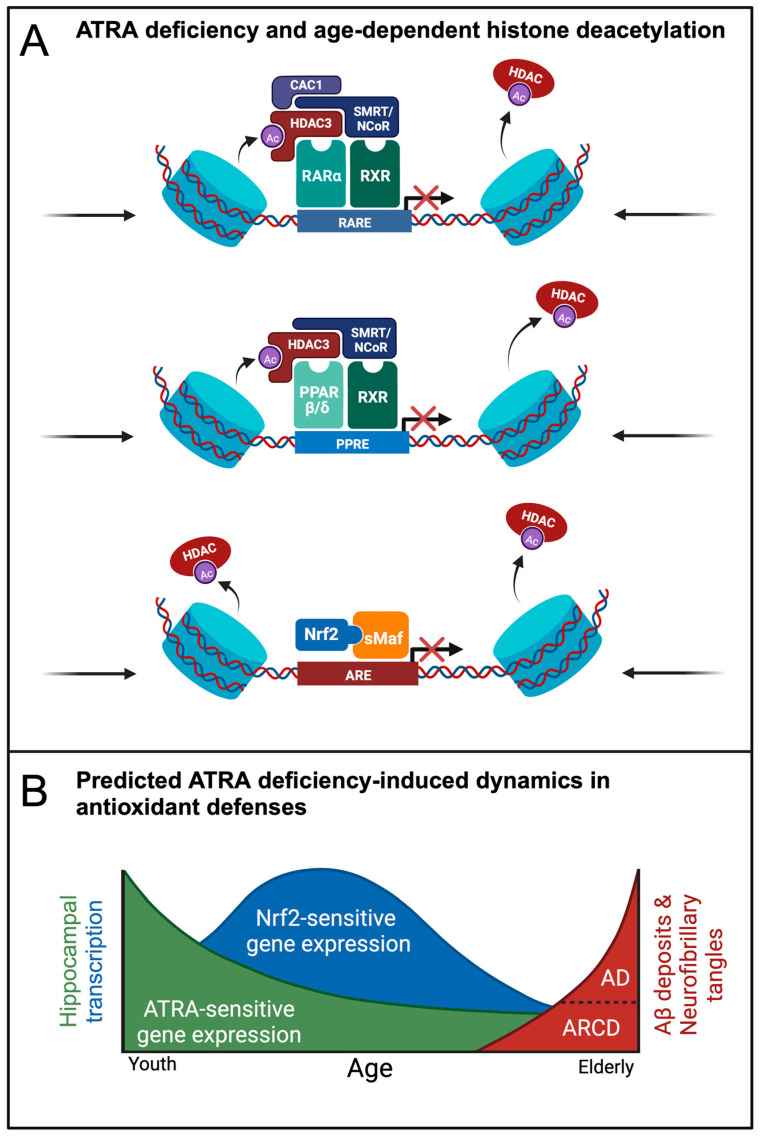
Proposed effects of age-related epigenetic changes in ATRA-sensitive and Nrf2-sensitive gene transcription, and AD progression. (**A**) ATRA deficiency is accompanied by a co-repressor complex that includes HDAC3. The accompanying age-related upregulation of class I/II HDACs and DMNTs further ensures transcriptional silencing of both ATRA- and Nrf2-sensitive genes. (**B**) The activity of ATRA as a dietary exogenous AO and transcriptionally active ligand is lost in the hippocampus, thereby increasing oxidative stress and Nrf2-mediated gene expression until epigenetic changes silence the transcription of Nrf2-driven pathways, resulting in expedited ARCD and AD progression.

We propose that the bioavailability of ATRA and endogenous AOs work in a coordinated fashion to maintain a homeostatic equilibrium of redox balance in the hippocampus. However, in aging and AD, this interplay between ATRA- and Nrf2-mediated regulation may be complicated by epigenetic changes that include changes in histone acetylation/deacetylation balance and RARE/PPARE/ARE related transcription, rendering RAREs, PPAREs, and AREs progressively inaccessible to the RAR-, PPAR- and/or Nrf2-mediated transcription needed to finely tune redox balance within memory circuits (Figure 4A). The eventual homeostatic collapse of this redox balance may hasten AD onset and progression (Figure 4B).

### 2.10. Dysregulation of Mitochondria-Related Genes Is Consistent with ATRA Depletion, Increased Oxidative Stress, and Mitochondrial Aβ Accumulation

A number of genes involved in mitochondrial (mt) function (TOMM20, TOMM40, OPA1, DNM1L) were significantly downregulated in AD in the human hippocampus (Table 3), suggestive of mt dysfunction induced by OS. Translocase of outer mitochondrial membrane 20 (TOMM20) and 40 (TOMM40) exist at the outer mitochondrial membrane and function to import mitochondrial preproteins synthesized within the cytosol [169,170,171]. Both TOMM20 and TOMM70 have previously been shown to be decreased in the AD hippocampus, indicative of mitochondrial respiration dysfunction [172,173]. The TOMM import machinery is also used in the translocation and accumulation of Aβ into the mitochondrial cristae, which is associated with synaptic deficits [174,175,176]. Therefore, the downregulation of the TOMM proteins may be attributed to active protective mechanisms in addition to transcriptional inactivity due to the absence of agonist. Optic atrophy 1 (OPA1) exists in the inner mitochondrial membrane (IMM) and normally functions in the fusion of the IMM [177]. OPA1 also plays a role in combating ROS accumulation and is similarly decreased in hippocampal AD brains [178,179]. 

Finally, dynamin-1-like protein (DNM1L; DRP1) is a GTPase that promotes mitochondrial fission, distribution of mitochondria throughout the neuron, neuronal differentiation, synapse formation, and dendrite formation [180,181,182]. ATRA has recently been shown to upregulate DNM1L protein expression [183]. Interestingly, DNM1L mRNA downregulation in the AD hippocampus is correlated with upregulation of retinoic acid receptor-related orphan receptor alpha (RORA) mRNA [184]. In accordance with this study, we observed that DNM1L mRNA was significantly downregulated and RORA mRNA upregulated in the human AD hippocampus (Table 3). The underlying molecular mechanisms are unclear because unlike RORβ, RORα is not activated by ATRA [185]. Furthermore, multiple studies allude to increased expression of DNM1L as a causative factor in AD pathogenesis due to increased mitochondrial fragmentation; beneficial effects of DNM1L inhibition have been observed in AD models [186,187,188,189]. Other studies have proposed that Aβ and phosphorylated tau interact abnormally with DNM1L, resulting in mitochondrial dysfunction and hastening AD progression [190,191,192,193,194,195,196,197,198]. Due to the integral role that DNM1L plays in normal neuronal function in the relative absence of Aβ and phosphorylated tau [199], ATRA may indirectly beneficially modulate DNM1L activity by α-secretase-mediated prevention of Aβ accumulation. Further investigation into the transcriptional control of DNM1L, the causes of its dysregulation, and its distinct roles in neuroprotection or neurotoxicity in AD and non-AD brains is needed before drawing definitive conclusions [200].

### 2.11. The Upregulation of Histone Deacetylases (HDACs) Suggests Epigenetic Changes That Interfere with RAR-and Nrf2-Mediated Transcription

Rates of transcription of ATRA-dependent genes involve mechanistic, epigenetic, and co-repressor factors. The inconsistency between the upregulation of Nrf2 mRNA expression and the downregulation of Nrf2-dependent gene targets (Table 3) led us to suspect that epigenetic mechanisms could play a role. Histone deacetylases (HDACs), together with histone acetyltransferases (HATs), set acetylation/deacetylation balance, allowing tight control over chromatin structure and gene transcription [201]. Among Class I HDACs, we found the most significant dysregulation in HDAC1 transcript from post-mortem AD hippocampus (Table 4). To a lesser extent, Class II HDACs, HDAC4 and HDAC7, were significantly upregulated. Together, we observed AD-related increases in HDAC expression, suggesting that relatively global epigenetic silencing of gene transcription underlies AD-related learning impairments. Therefore, even if ATRA were to be locally present at sufficient levels, histone deacetylation may preserve the closed chromatin conformation and promoter inaccessibility that downregulate critical ATRA- and Nrf2-induced gene expression. The finding of HDAC upregulation across class I/II HDACs suggests that a pan-HDAC inhibitor may counteract epigenetic changes, offering hope in treating AD onset and progression [202,203]. Indeed, HDAC inhibition has shown promising effects in AD mouse models [203,204].

### 2.12. Age-Related Epigenetic Changes Associated with RARE, PPARE, and ARE

Histones 3 and 4 (H3 and H4, respectively) have been implicated in the regulation of Nrf2 gene expression. H4 acetylation has been shown to increase transcription of Nrf2-dependent genes [205], while H3 and H4 deacetylation have been shown to reduce Nrf2 gene expression in microglia [206]. H3 acetylation is associated with unmethylated CpG islands in the Nrf2 promoter [207]. These results suggest that a HDAC inhibitor may optimally restore repressed Nrf2 expression. However, this therapeutic intervention only accounts for Nrf2 gene silencing attributed to epigenetic changes and does not account for Nrf2 inhibition or downregulation attributed to dysregulation of Nrf2 at the protein-protein interaction or transcription factor levels of regulation, such as by Keap1, GSK-3β, Bach1, p53, Hrd1, and miRNAs [208,209].

In addition to the decline in hippocampal ATRA levels due to age-related homeostatic collapse of the liver-brain axis [57], AD-related epigenetic changes may directly interfere with RAR- and PPAR-mediated transcriptional activity. In post-mortem AD brains and mouse models, histone deacetylation has been heavily implicated in the progression of AD [210,211,212,213,214,215]. Furthermore, VA deficient mice have lower levels of histone acetylation due to dysregulation of CBP-dependent mechanisms [216]. The therapeutic employment of class I/II HDAC inhibitors has shown wide-spread promise in treating AD by improving learning, memory, and behavioral functions, reducing neuroinflammation, and decreasing levels of Aβ and phosphorylated tau [203,204,217,218,219,220,221,222,223,224,225,226,227]. 

**Figure 5:**
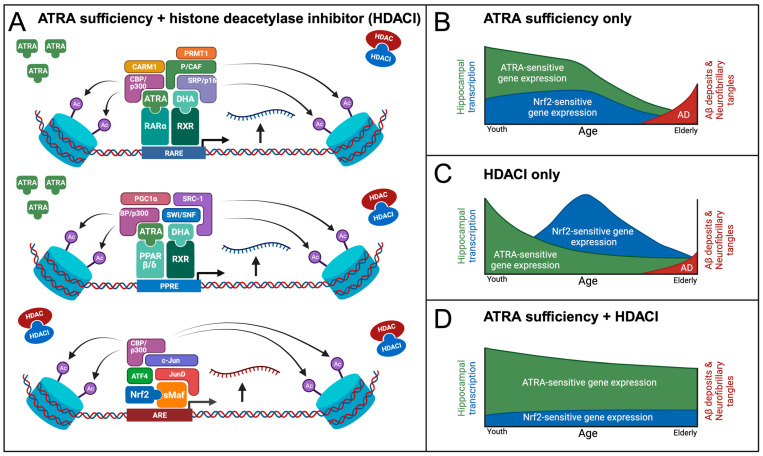
Working model of therapeutic effects of HDAC inhibitors on the activation of RAR-, PPAR-, and Nrf2-mediated gene transcription. (**A**) ATRA sufficiency removes the co-repressor complex and recruits histone acetylases, histone methyltransferases, and transcription factors. Therapeutic employment of a class I/II HDAC inhibitor results in an open chromatin conformation and accessible promotor region that augments the beneficial effects of ATRA sufficiency. Also, Nrf2 is transcriptionally active in circumstances of ATRA deficiency combined with a HDAC inhibitor. (**B**) Sufficient hippocampal ATRA concentration staves off AD progression until age-related epigenetic changes silence ATRA- and Nrf2-sensitive gene expression. (**C**) With ATRA insufficiency, epigenetic intervention alone will increase compensatory Nrf2-mediated AO defenses, aiding in delaying MCI and AD, but ATRA-deficiency remains, preventing the expression of ATRA-induced neuroprotective pathways. (**D**) HDAC inhibition, in combination with retinoid supplementation, lifts the epigenetic silencing of RAREs, PPAREs, and AREs, and allows for the activation of both ATRA- and Nrf2-responsive neuroprotective genes.

On the basis of these observations, we propose the following working model. Under conditions of ATRA deficiency, the unliganded RAR/RXR heterodimer binds to HDAC3 via co-repressors nuclear receptor corepressor (NCOR) or Silencing Mediator of Retinoid and Thyroid Hormone Receptors (SMRT) as well as corepressor CDK2-associated cullin domain 1 (CACUL1) [228,229,230]. HDAC3 functions by deacetylating nearby histones, further ensuring transcriptional inactivity of ATRA- and PPAR-sensitive genes [231] (Figure 4A). In the presence of ATRA, the liganded RAR/RXR heterodimer complex binds to histone acetyltransferases (HATs) such as CREB binding protein/p300 (CBP/p300), steroid receptor coactivator/p160 (SRC/p160), and p300/CBP-associated factor (P/CAF) (Figure 5A). Acetylation of histones by HATs promotes an open chromatin structure, increasing transcription. Histone methyltransferases (HMTs) such as coactivator associated arginine methyltransferase 1 (CARM1) and protein arginine methyltransferase 1 (PRMT1) are also recruited, responsible for methylating arginine residues on histones that can further activate the complex and increase gene expression [232], although histone methylation can also be silencing, with the effects depending on context and methylation pattern [233]. The association of PRMT1 with a RAR/RXR heterodimer bound to a RARE allows for a localized open chromatin conformation and subsequent transcription of ATRA-sensitive genes (Figure 5A).

ATRA depletion is also predicted to negatively affect PPARβ/δ-mediated gene expression. Unliganded RARα recruits NCOR or SMRT, decreasing ATRA-PPARβ/δ gene transcription [234] (Figure 4A). Similarly to RARα, these co-repressors are removed with the binding of ATRA to PPARβ/δ. Subsequent heterodimerization with RXR recruits numerous co-activators: peroxisome proliferator-activated receptor-γ coactivator 1-α (PGC1α), switch/sucrose non-fermentable (SWI/SNF), CBP/p300, and steroid receptor coactivator 1 (SRC-1), the latter two of which are HATs [235,236]. The recruitment of these transcriptional co-activators and histone acetyltransferases increases ATRA-PPARβ/δ-RXR-sensitive gene expression, ultimately enhancing neuroprotection against inflammation and OS (Figure 5A). 

Finally, when ATRA is present and bound to RARα, the complex binds to the Neh7 region on Nrf2, sequestering bioavailable Nrf2 and functioning as an ARE-bound co-repressor. We hypothesize that ATRA depletion, as well as depletion of other antioxidants, causes an increase in OS due to the lack of exogenous AO effect, resulting in the activation of compensatory Nrf2-mediated endogenous AO defenses (Figure 3B). However, histone deacetylation may progressively reduce Nrf2-sensitive gene expression, despite elevated OS. We propose a model in which the degree to which ATRA-RARα-Nrf2 sequestration occurs is contingent on cellular concentrations of ATRA. Under this model, excessive levels of ATRA lead to Nrf2 sequestration, but moderate ATRA levels do not, allowing Nrf2 to exert its homeostatic effects by recruiting c-Jun, JunD, activating transcription factor 4 (ATF4), and the HAT CBP/p300. It has been shown that CBP/p300 not only acetylates nearby histones to promote an open chromatin conformation, but also directly acetylates Nrf2 to augment the formation of the transcriptional complex [237]. By recruiting these transcription factors, there is increased gene expression of endogenous AOs such as NQO1/2, HO-1, GSTM4, and SOD1, providing neuroprotection that counteracts OS and attenuates the neurodegenerative process of AD [238] (Figure 5D).

## 3. Limitations, Open Questions, and Future Directions

Although we cover a variety of mechanistic aspects, there are a number of limitations associated with our assessment of human hippocampal transcriptomics and open questions remaining. 

First, as we solely focused on the human dataset originally created by van Rooij and colleagues [107], we have not incorporated additional human datasets to compare and validate specific genes and RAR/PPARδ/β signaling pathways. Moreover, this data represents a snapshot in time during which AD has significantly progressed (Braak stage 5.5). The examination of the human hippocampal transcriptome earlier in AD pathogenesis (i.e., in mild cognitive impairment and/or earlier Braak stages) in the future will provide additional insights on the temporal relationship of VA status with respect to AD pathogenesis and progression. Nevertheless, the van Rooij et al. human dataset was adequately powered, statistically sound, and was highly heterogeneous, including both sexes and a mixture of APOE4 phenotypes. Therefore, observing these robust effects for the genes examined, even in the face of considerable heterogeneity, suggests that the involvement of VA mechanisms and ATRA-sensitive genes could be generalizable to additional time points and datasets.

Second, transcriptomics data is invaluable in providing data on thousands of mRNA transcripts; however, it is important to remember that gene expression may not perfectly correlate with protein expression. We assume that the binding of RARα/RXR to RAREs increases gene transcription at RARE sites on the promoter region of any given ATRA-sensitive gene (Figure 2), but this is most likely an oversimplification, as this is not always the case for any given ATRA-sensitive gene. We are acutely aware that additional bioinformatics resources exist on ATRA-sensitive gene expression. Several databases, such as TF2DNA and Enrichr, can provide detailed information on potential RAREs/PPAREs associated with specific genes. This work is limited in that it does not incorporate all of the available resources on ATRA-sensitive gene expression. A brief foray into Enrichr for any given ATRA-sensitive gene reveals a seemingly overwhelming number of additional transcription factors that may or may not be active with RARα-mediated gene transcription at RAREs, implying a complexity that is not yet well understood. Moreover, this study is based solely on RNA-seq data, which lacks cell type-specific information. We suspect that downregulation of ATRA-dependent mRNA transcripts is likely to occur in neuronal populations, whereas upregulation of ATRA-dependent mRNA transcripts could involve non-neuronal populations that relate to neuroinflammatory signaling mechanisms. Therefore, much work remains in determining how ATRA-sensitive gene expression in specific neuronal cell types is impacted by global ATRA deficiency that may be present in AD pathology.

Third, it is important to remember that VA plays important roles not only in the brain but also in other organ systems. A landmark rodent study demonstrating that age-related VA dysregulation involves disruption of the liver-brain axis [57] is a reminder that our understanding of the liver-brain axis in humans is at a nascent stage. The functional roles of retinoids in the brain could be impaired by insufficient dietary intake of VA, defective hepatic storage of VA, impaired hepatic release of retinol and RBP4, impaired transport of retinol-RBP4-TTR from liver to brain, and/or defective processing of retinol into ATRA in the human hippocampus [98]. In this study, we showed that there is a highly significant downregulation of hippocampal RBP4 (Table 1), which suggests impaired vascular transport of retinol from the liver to distant tissues, including the brain, assuming that hippocampal RBP4 downregulation is reflective of a similar downregulation in the liver. Although further study is needed to explore this possibility; such a finding is consistent with hepatic trapping of VA and dysregulation of the brain-liver axis as a potential upstream mechanism of ATRA deficiency in AD hippocampus. Therefore, it is likely that VA deficiency in brain is preceded by liver damage and hepatic trapping of VA stores in liver. Malabsorption of VA in gut, as well as dysbiosis, could contribute to early stages of disease pathogenesis, inducing local ATRA deficiency and inflammatory signaling. There is no doubt that co-morbidities occurring in midlife may increase the risk of AD-related dementia later in life. Therefore, a greater understanding of VA dysregulation in other disease processes (i.e., non-alcoholic fatty liver disease, cardiovascular dysfunction, obesity, diabetes) may provide important key insights into AD pathogenesis. These areas of research are exciting future directions but fall beyond the scope of this review.

Fourth, ATRA has many pleiotropic functions in the CNS. Not only is it considered an endogenous antioxidant with free radical scavenging capabilities like retinol and carotenoids [140,141,142,239], it is also a receptor ligand for RARs and PPARδ/β [31,139,240]. However, in addition to acting as hormone-like transcription factors at the genomic level, RARs also are known to exhibit non-genomic actions [79,241,242]. At a synaptic level, ATRA balances excitatory and inhibitory synaptic strength as part of its role in homeostatic plasticity and in preventing runaway Hebbian synaptic plasticity [242]. ATRA has also been shown to contribute to the cellular regulation of kinases and protein translation [164]. Moreover, ATRA synthesis appears to be tightly controlled by intracellular calcium dynamics [243]. Finally, ATRA plays an important role in neurogenesis, but these actions may be, in part, mediated by RAR-independent, non-genomic actions of ATRA [244], which have implications for neurodegenerative diseases [245]. These non-genomic mechanisms of ATRA are interesting and relevant to Alzheimer’s disease pathogenesis. We have limited this review to focus on the genomic actions of ATRA. However, it is likely that ATRA deficiency in AD would alter non-genomic aspects of ATRA signaling. These are interesting directions that can be examined in the future.

Fifth, although our foray through ATRA-sensitive genes and RAR co-repressors make a good case for ATRA deficiency, not all ATRA-sensitive genes exhibit downregulation consistent with ATRA deficiency. To this point, our initial examination of ATRA-sensitive transcripts does not directly support changes in the balance between amyloidogenic vs. non-amyloidogenic pathways in human AD. In this human dataset, based on previous studies (see above), we had predicted that the most critical of ATRA-sensitive genes to be the α-secretase ADAM10. Under amyloidogenic conditions, we predicted that ADAM10 mRNA would be downregulated relative to BACE1 mRNA, setting the stage for amyloidogenic pathway activation, accounting for AD progression. Surprisingly, we found ADAM10 mRNA to be significantly upregulated relative to BACE1 in this AD dataset (Table 5). As there is a known RARE within the ADAM10 promoter, this observation is inconsistent with a simple interpretation of ATRA deficiency. However, both ADAM10 and BACE1 had fairly weak DE scores (0.03 and 0.01, respectively), raising the question as to whether additional ADAM family members and/or BACE genes may contribute (Table 5). Given the constraint that we are primarily examining late-stage AD, additional compensatory mechanisms may be at play that complicate the interpretation of ADAM10 mRNA expression. We suspect that alternative transcriptional activators of ADAM10, such as PPARα, and especially SIRT1 [29,47,246], may partly account for this discrepancy, since both transcripts are significantly upregulated in this AD dataset (Table 5). Additional proteolytic ADAM family metalloproteinases are detected in this dataset (i.e., ADAM8, ADAM17, ADAM33), but these are also significantly upregulated similarly to ADAM10. It is possible that there are additional unknown compensatory mechanisms that come into play in an attempt to restore homeostasis. Finally, transcriptional upregulation may be a compensatory mechanism and may not necessarily translate to protein expression, giving no insight into potential downstream mechanisms. Further analysis, using additional datasets, and especially earlier timepoints, is necessary to interpret the broader meaning of this finding. That this simple prediction fails suggests that intrinsic transcriptional mechanisms are far more complex and will take more time and effort to unravel the underlying molecular mechanisms.

Sixth, HDAC inhibitors are not the only useful agents in reversing age-related epigenetic changes. For example, sulforaphane, a DNA methyltrasferase (DNMT) inhibitor found in cruciferous vegetables, upregulates Nrf2 expression by decreasing the methylation of cytosine residues in promoter CpG dinucleotides, lifting the epigenetic suppression of Nrf2 as well as many other genes. This action was also found to decrease levels of Aβ, ROS, and neuroinflammation in mouse models [247]. Similarly, resveratrol downregulates the expression of DNMTs, which decreases methylation at the Nrf2 promoter site. This increases Nrf2 expression and decreases levels of OS [248]. Interestingly, resveratrol has also been shown to reverse ATRA resistance by demethylating the CRABP2 gene, responsible for nuclear import of ATRA and its subsequent transfer to RARα [249]. There is even some evidence that the herbal supplement curcumin and exercise may also contribute to the demethylation of CpG islands in the promoter site of the Nrf2 gene and increase transcriptional expression [250,251]. Therefore, the incorporation of epigenetic changes into our working model is far from complete, with future drug combinations between nuclear receptor ligands, HDAC inhibitors, and DNMT inhibitors worthwhile to explore in future studies.

Finally, SIRT1, a class III HDAC, has been proposed to be neuroprotective against AD by deacetylating CRABP2 and RARβ, resulting in increased expression of α-secretase and reducing amyloidogenesis [252,253,254]. Moreover, it has even been proposed that SIRT1 has a direct agonist effect on RARβ that activates transcription of α-secretase [255]. Despite a previous retraction [256], this area of research has been extremely active. However, because these mechanisms likely involve DNA hypermethylation [257,258,259,260,261,262,263,264], we feel that these areas are beyond the scope of this review and best saved for a future article dedicated to this focus.

## 4. Conclusions

This is the first review to explicitly discuss ATRA-sensitive genes within the context of a human hippocampal transcriptomics dataset. We have explored possible molecular mechanisms stemming from select dysregulated genes in a publicly available hippocampal transcriptomic dataset from postmortem AD and control brains [107]. First, we demonstrate that a number of genes mediating retinol transport, ATRA synthesis, and ATRA metabolism are dysregulated in hippocampal tissue from post-mortem AD brains, supporting the hypothesis that ATRA deficiency occurs in the human hippocampus in AD. Second, we discovered transcriptional upregulation of RAR-related co-repressor genes, corroborating the idea of transcriptional repression of RAR-mediated transcription. Third, we noted increased expression of OS and neuroinflammatory genes is consistent with ATRA depletion. Interestingly, we encountered evidence from the cancer field of crosstalk between Nrf2- and RAR-mediated signaling, supporting the idea that oxidative stress-mediated Nrf2 upregulation is consistent with depletion of VA and other lipophilic vitamins (i.e., E and K) thought to have ROS scavenging activity. Moreover, increased NFKB1 and NFKB2 transcripts are associated with neuroinflammation which may be caused in part by oxidative stress. Interestingly, we noted that many targets of Nrf2 were not upregulated (Table 3), suggesting an inability of Nrf2 to modulate transcription. These observations raised the possibility that HDAC upregulation (Table 4) may partly account for the failure of Nrf2 targets to respond to Nrf2 upregulation. 

Based on these observations, we have developed a working model of transcriptional mechanisms in AD, centering on ATRA deficiency and age-dependent epigenetic silencing (Figure 5). We propose that VA sufficiency combined with HDAC inhibition could sustain cognition and potentially avoid AD. Interestingly, we found sparse evidence for downregulation of ADAM10 in AD, as expected from ATRA deficiency (Table 5). However, given the limitations stated above, potential compensation effects late in the disease process may explain this observation, as well as other ADAM family members that may complicate this interpretation. Alternatively, our findings suggest renewed appreciation in ATRA as a ROS scavenger and the role of antioxidant depletion, generally, in causing oxidative stress and triggering of antioxidant defenses. Nevertheless, we feel that disruption of the balance between non-amyloidogenic and amyloidogenic pathways is an essential component of AD and should still be considered as a working model of AD (Figure 1). Furthermore, since ATRA binds to PPARδ/β receptors with high affinity [31], targets of PPARδ/β signaling also would be dysregulated, further contributing to OS elevation, neuroinflammation, Aβ plaque formation, and phosphorylated tau, which together accelerate AD onset and progression [51]. Lastly, we predict that Nrf2-mediated endogenous AOs are indeed engaged early in AD to combat the rising ROS. However, increased expression of HDACs and aberrant epigenetic silencing may explain global downregulation of RAR- and Nrf2-dependent genes in AD.

Overall, our mechanistic insights point towards VA supplementation, combined with HDAC inhibition, as a plausible AD prevention strategy (Figure 5D). Although several HDAC inhibitors are approved for use as drugs (i.e., vorinostat), a growing number of naturally occurring active metabolites in food are appreciated as HDAC inhibitors [265,266], many of which are associated with Mediterranean diet and healthy aging. Therefore, it is possible that a particular combination of nutrients, including not only VA sufficiency but also HDAC inhibition across lifespan, promotes healthy aging, longevity, and AD prevention.

## Figures and Tables

**Table 1:** Evidence for transcriptional dysregulation of ATRA transport, synthesis, and metabolism in the hippocampus of human AD. Downregulated mRNA transcripts are shown in blue, upregulated mRNA transcripts are shown in red, and transcripts that show no significant change are in shown in gray. Each gene includes log fold-change (logFC), *p* value, false discovery rate (FDR), and differential expression (DE) score, as performed by van Rooij and colleagues [107].

Gene	Function	logFC	*p* Value	FDR	DE Score
RBP4	Retinol transport	−1.30	7.9 × 10^−13^	3.4 × 10^−11^	0.43
TTR	Retinol transport	0.57	1.7 × 10^−1^	2.6 × 10^−1^	0.01
STRA6	Retinol neuronal import	−0.72	6.4 × 10^−2^	1.1 × 10^−1^	0.02
RDH12	Retinal synthesis	−0.92	9.8 × 10^−4^	3.2 × 10^−3^	0.08
RBP1	Retinol transport	−0.78	3.2 × 10^−9^	4.7 × 10^−8^	0.19
ADLH1A1	ATRA synthesis	−0.18	3.2 × 10^−1^	4.2 × 10^−1^	0.00
ADLH1A2	ATRA synthesis	−0.47	1.2 × 10^−1^	1.9 × 10^−1^	0.01
ADLH1A3	ATRA synthesis	−0.71	5.0 × 10^−5^	2.3 × 10^−4^	0.09
CRABP1	Nuclear translation	0.38	3.1 × 10^−1^	4.1 × 10^−1^	0.00
CRABP2	Nuclear translocation	−0.41	9.6 × 10^−2^	1.6 × 10^−1^	0.01
FABP5	Nuclear translocation	−0.19	2.5 × 10^−1^	3.4 × 10^−1^	0.00
CYP26A1	ATRA-sensitive ATRA catabolism	−1.09	6.7 × 10^−3^	1.7 × 10^−2^	0.06
CYP26B1	ATRA-sensitive ATRA catabolism	−0.97	2.5 × 10^−6^	1.7 × 10^−5^	0.15
RARA	α retinoic acid receptor	−0.05	7.4 × 10^−1^	8.1 × 10^−1^	0.00
RARB	β retinoic acid receptor	0.25	7.2 × 10^−2^	1.3 × 10^−1^	0.01
RARG	γ retinoic acid receptor	0.68	6.3 × 10^−7^	4.9 × 10^−6^	0.12
SP1	RARG transcriptional regulator	0.63	1.0 × 10^−8^	1.3 × 10^−7^	0.14
LRAT	ATRA into AT-retinyl ester	0.61	1.2 × 10^−2^	2.7 × 10^−2^	0.03

**Table 2:** Evidence for transcriptional block of ATRA signaling in the hippocampus of human AD. Red highlighting indicates upregulated mRNA transcripts. Each gene includes log fold-change (logFC), *p* value, false discovery rate (FDR), and DE score, as performed by van Rooij and colleagues [107].

Gene	Function	logFC	*p* Value	FDR	DE Score
**NCOR1**	Class II nuclear receptor co-repressor	0.32	9.5 × 10^−4^	3.1 × 10^−3^	0.03
**NCOR2**	Class II nuclear receptor co-repressor	0.10	2.6 × 10^−1^	3.5 × 10^−1^	0.00
**ZBTB16**	Class II nuclear receptor co-repressor	0.96	3.5 × 10^−9^	5.0 × 10^−8^	0.23
**TNIP1**	RAR co-repressor	0.35	5.9 × 10^−6^	3.6 × 10^−5^	0.05
**RIF1**	RAR co-repressor	0.23	2.6 × 10^−2^	5.2 × 10^−2^	0.01
**LCOR**	Class I/II nuclear receptor co-repressor	0.49	7.7 × 10^−4^	2.6 × 10^−3^	0.04

**Table 3:** Indirect evidence for hippocampal ATRA depletion-induced increases in oxidative stress, inflammation, and mitochondrial dysfunction in human AD. Downregulated mRNA levels are shown in blue; upregulated mRNA levels are shown in red. Each gene includes log fold-change (logFC), *p* value, false discovery rate (FDR), and DE score, as performed by van Rooij and colleagues [107].

Gene	Function	logFC	*p * Value	FDR	DE Score
**NFE2L1**	ROS sensor-transcription factor	0.54	2.0 × 10^−10^	3.9 × 10^−9^	0.15
**NFE2L2**	ROS sensor-transcription factor	0.46	1.0 × 10^−4^	4.5 × 10^−4^	0.05
**NFKB1**	Inflammation	0.80	2.6 × 10^−14^	1.9 × 10^−12^	0.27
**NFKB2**	Inflammation	0.64	1.1 × 10^−7^	1.1 × 10^−6^	0.13
**NQO1**	Endogenous AO	−0.22	4.2 × 10^−1^	5.2 × 10^−1^	0.00
**NQO2**	Endogenous AO	−0.36	2.7 × 10^−3^	7.7 × 10^−3^	0.03
**GSR**	Endogenous AO	−0.25	2.5 × 10^−3^	7.2 × 10^−3^	0.02
**GSTA4**	Endogenous AO	−0.92	7.6 × 10^−16^	1.1 × 10^−13^	0.31
**GSTM4**	Endogenous AO	−0.39	7.1 × 10^−4^	2.4 × 10^−3^	0.03
**GSTO1**	Endogenous AO	−0.32	3.4 × 10^−3^	9.2 × 10^−3^	0.02
**GSTO2**	Endogenous AO	−0.47	8.0 × 10^−3^	1.9 × 10^−2^	0.03
**GSTZ1**	Endogenous AO	−0.80	5.0 × 10^−5^	2.4 × 10^−4^	0.10
**SLC7A11**	Cysteine/glutamate transporter	−0.26	6.4 × 10^−2^	1.1 × 10^−1^	0.01
**PTGR1**	Endogenous AO	−0.59	1.3 × 10^−4^	5.3 × 10^−4^	0.06
**HMOX2**	Endogenous AO	−0.55	4.5 × 10^−8^	4.7 × 10^−7^	0.12
**SOD1**	Endogenous AO	−0.31	2.8 × 10^−2^	5.7 × 10^−2^	0.01
**GLRX3**	Endogenous AO	−0.16	4.7 × 10^−2^	8.7 × 10^−2^	0.01
**CAT**	Endogenous AO	0.42	1.6 × 10^−3^	4.9 × 10^−3^	0.03
**TOMM20**	Protein targeting to mitochondria	−0.61	3.1 × 10^−11^	7.8 × 10^−10^	0.19
**TOMM40**	Protein targeting to mitochondria	−0.56	1.8 × 10^−8^	2.1 × 10^−7^	0.12
**OPA1**	Regulates mitochondrial fusion	−0.81	1.9 × 10^−14^	1.5 × 10^−12^	0.27
**DNM1L**	Regulates mitochondrial fission	−0.79	1.3 × 10^−13^	7.2 × 10^−12^	0.26
**RORA**	Retinoic acid-related orphan receptor α	0.48	3.2 × 10^−4^	1.2 × 10^−3^	0.05

**Table 4:** Indirect evidence for transcriptional dysregulation via upregulation of histone deacetylases (HDACs) in human AD. Upregulated mRNA levels are shown in red. Each gene includes log fold-change (logFC), *p* value, false discovery rate (FDR), and DE score.

Gene	Function	logFC	*p* Value	FDR	DE Score
**HDAC1**	Class I histone deacetylase	0.50	8.3 × 10^−8^	8.1 × 10^−7^	0.10
**HDAC2**	Class I histone deacetylase	0.09	2.7 × 10^−1^	3.7 × 10^−1^	0.00
**HDAC4**	Class II histone deacetylase	0.26	1.2 × 10^−2^	2.8 × 10^−2^	0.01
**HDAC5**	Class II histone deacetylase	0.14	9.1 × 10^−2^	1.5 × 10^−1^	0.00
**HDAC7**	Class II histone deacetylase	0.66	1.4 × 10^−9^	7.7 × 10^−3^	0.03

**Table 5:** ADAM- and BACE-family members in human AD. Upregulated mRNA levels are shown in red. Each gene includes log fold-change (logFC), *p* value, false discovery rate (FDR), and DE score.

Gene	Function	logFC	*p* Value	FDR	DE Score
**ADAM10**	proteolytic α-secretase	0.34	2.9 × 10^−4^	1.1 × 10^−3^	0.03
**PPARα**	ADAM10 activator	0.37	6.7 × 10^−3^	1.7 × 10^−2^	0.02
**SIRT1**	ADAM10 activator	0.77	1.8 × 10^−10^	3.7 × 10^−9^	0.22
**BACE1**	β-secretase	−0.19	9.3 × 10^−2^	1.6 × 10^−1^	0.01
**ADAM8**	proteolytic α-secretase	0.63	7.5 × 10^−3^	1.8 × 10^−2^	0.04
**ADAM9**	proteolytic α-secretase	0.18	1.2 × 10^−1^	1.9 × 10^−1^	0.00
**ADAM15**	proteolytic α-secretase	−0.06	6.7 × 10^−1^	7.5 × 10^−1^	0.00
**ADAM12**	proteolytic α-secretase	0.44	3.9 × 10^−2^	7.5 × 10^−2^	0.02
**ADAM17**	proteolytic α-secretase	0.37	1.3 × 10^−3^	3.9 × 10^−3^	0.03
**ADAM19**	proteolytic α-secretase	0.37	7.4 × 10^−2^	1.3 × 10^−1^	0.01
**ADAM33**	proteolytic α-secretase	1.55	8.3 × 10^−12^	2.6 × 10^−10^	0.49
**ADAM11**	non-proteolytic ADAM	−1.36	5.5 × 10^−9^	7.4 × 10^−8^	0.32
**BACE2**	neuroprotective β-secretase	1.63	1.1 × 10^−11^	3.2 × 10^−10^	0.52

## Data Availability

All data discussed are available via Supplementary Materials by van Rooij and colleagues [107].
